# Quality of life, psychological distress and violence among women in close relationships: a population-based study in Finland

**DOI:** 10.1186/s12905-020-00950-6

**Published:** 2020-04-28

**Authors:** Tomomi Hisasue, Marie Kruse, Jani Raitanen, Eija Paavilainen, Pekka Rissanen

**Affiliations:** 1 Faculty of Social Sciences (Health Sciences), Tampere University, FI-33014 Tampere, Finland; 2 Finnish Institute for Health and Welfare, P.O. Box 30, FI-00271 Helsinki, Finland; 3 Danish Centre for Health Economics, University of Southern Denmark, J. B. Winsløwsvej 9B, 1, DK-5000 Odense C, Denmark; 4 UKK Institute for Health Promotion Research, P.O. Box 30, 33501 Tampere, Finland; 5 Southern Ostrobothnia Hospital District, Hanneksenrinne 7, 60220 Seinäjoki, Finland

**Keywords:** Violence, Quality of life, Psychological distress, EUROHIS-QOL 8-item index, Finland

## Abstract

**Background:**

The aim of this study was to examine associations between exposure to violence, quality of life, and psychological distress. Women aged 19–54 years who had been exposed to violence by someone in a close relationship were compared with women unexposed to violence in Finland. We also aimed to investigate associations between different forms of violence (physical, sexual, emotional, or any combination of these) with quality of life and psychological distress.

**Methods:**

We selected a sample of 22,398 women who had returned self-completed questionnaires from a Finnish population-based health survey between 2013 and 2016. Exposure to violence during the past year was assessed through specific questions from the survey. The EUROHIS-QOL 8-item index was used to measure quality of life, and ordinary least square regressions were fitted. The mental health inventory (MHI-5) was used to measure psychological distress. We investigated associations with multivariate logistic regression analysis.

**Results:**

Among women in Finland, the prevalence of exposure to violence in any type of close relationship during the past year was 7.6%. Women who had been exposed to violence had significantly worse scores of the EUROHIS-QOL 8-item index, and psychological distress was significantly worse (*p* < 0.001), compared with unexposed women. Strong associations were found between combinations of violence and both quality of life (coefficient − 0.51, *p* < 0.001) and mental health (odds ratio 4.16, 95% confidence interval 3.44–5.03). Compared with women who had been exposed to violence by a stranger, women who had been exposed to violence by someone in a close relationship had significantly lower quality-of-life scores (*p* < 0.001).

**Conclusions:**

This study found that experience of close relationship violence had a negative influence on both quality of life and psychological distress among women in the general Finnish population. Comparison with victims of violence by strangers shows that some of the lower quality-of-life scores among victims are driven by the perpetrator and victim being in a close relationship. Preventive policies in primary care settings aimed at screening and educating young people should be considered as an early form of intervention to reduce the negative mental health consequences of violence.

## Background

Violence in close relationships is a widespread, serious public health problem worldwide [1]. The term “violence in close relationships” has been used in the Nordic countries and includes “intimate partner violence (IPV)”, “domestic violence”, and “family violence” in different contexts [2, 3]. It encompasses different forms of physical, sexual, and emotional violence and controlling behaviours by a perpetrator well known to the victim, e.g. a partner, ex-partner, sibling, or parents. Children witnessing domestic violence are also victims of violence in close relationships. Although both men and women are exposed to violence in close relationships, most often the victims are women [4]. Exposure to violence is associated with deteriorations in both short- and long-term health and well-being, as well as with increased risk of suicide, physical injuries, and long-term psychological effects—not only among victims [5] but also among their children [6, 7]. The negative health consequences of violence among victims may persist even after the violence has ended [8, 9].

There is a growing body of literature that uses of Quality of Life (QoL) as an outcome measure to assess subjective well-being and examine the impact of different health conditions among populations [10]. Despite this, studies on the consequences of violence in close relationships for QoL are scarce. Previous studies with relatively small population sizes in clinical settings [11, 12] or shelters [13] have shown associations between violence and poorer QoL, notably in areas of mental health and role- or social functioning [12, 13]. However, these findings may only capture the effects on QoL and violence in more severe cases where victims had access to public services or otherwise had the opportunity to disclose their exposure to violence.

A challenge when conducting research among victims of violence is the assumed underreporting of violence [14]. Only a few studies have used a national survey to examine QoL and violence in the general population. A Danish study found negative effects of recent physical violence on QoL [15], whereas the associations between other types of violence and QoL in the general population have remained poorly recognised.

Despite the scarcity of evidence on violence and QoL, it seems to be growing in relation to the consequences of different types of violence for mental health [16]. However, merely focusing on the diagnostics and treatment of mental health symptoms could be preventing health professionals from fully grasping the role of violence in the life of its victims. In addition, treating victims as mental health patients can reduce the likelihood of violence victims disclosing their experiences of violence [17].

Hence, investigation of possible associations between violence exposure in close relationships and QoL, and between mental health and violence, is needed.

The Finnish national health and well-being population-based survey (Regional Health and Well-being Study (ATH)) has been undertaken annually since 2010 [18]. A recent Finnish study using the same survey highlighted that alcohol abuse, psychological distress, and suicidal thoughts were associated with exposure to violence by a current partner among violence victims who have children under 18 years of age [19]. Conversely, the association between close-relationship violence against women and QoL, as well as psychological distress, has not been explored in a population-based setting.

The ATH survey included questions relating to exposure to violence and a generic QoL instrument (EUROHIS-QOL 8-item index) [20], which has not previously been used for victims of violence. As some of the shorter generic measures of QoL might have limitations in capturing some mental health impacts or environmental factors affecting health [21], a combination of measures covering generic QoL and mental health status using valid instruments could help capture associations between well-being and violence, and might be relevant for evaluating violence [22]. We assessed recent violence exposure over the past 12 months instead of lifetime exposure, as the former has stronger effects on mental health and/or well-being [11, 23]. Further, in the longer term, recall bias may mean that the validity of reports of violence exposure in the distant past is poorer than that relating to violence within the past year.

Adult male victims in close relationships are often reluctant to report their violence experience [24] and usually have minor injuries compared to women [25]. According to a systematic review of the literature [26], even though victimisation rates among women and men are similar, a number of studies have shown that women exposed to domestic violence are more likely than men to suffer from anxiety and depression. Similarly, in a study on men’s experiences of violence in Finland, although the frequency of exposure in close partner relationships was similar between the two genders, women experienced more serious mental consequences than did men [27]. However, research related to the psychological effects on men is sparse. Although the gender disparities are an important issue, our focus here is adult female victims of various forms of violence in close relationships, and men were excluded from the study.

The aim of this study was to examine the association between exposure to violence and QoL and psychological distress by comparing female respondents with and without exposure to violence in close relationships in the general adult population aged 19–54 years. Our definition of violence in close relationships included “domestic violence”, “intimate partner violence”, and violence perpetrated by someone well-known to the victim. By quantifying the association between QoL and violence, we aimed to focus on the complexities of close-relationship violence as a public health concern.

Our hypothesis was that women who have been exposed to violence, regardless of the type of violence, would have worse QoL and higher psychological distress than women unexposed to violence. We hypothesised that all types of violence have similarly negative associations with both QoL and psychological distress.

## Methods

Our data were collected from ATH surveys carried out from 2013 to 2016. The ATH survey is a Finnish nationally representative, cross-sectional, self-administered questionnaire (postal or online) based health survey of the Finnish population. A random sample stratified by age and regions across the country was drawn from the Finnish Population Register [18]. The invitation letter included information on the purposes of the survey, as well as information on data security and the use of data for health-monitoring and research purposes. A total of 169,500 individuals responded (response rate 54%) in 2013–15, and a sample of 5000 individuals (response rate 50%) responded in 2016 [28]. The study sample did not include the same individuals from different years. Women aged 19–54 years who responded to the surveys were selected for this study (*n* = 22,398).

### Outcomes

#### Quality of life measures

The EUROHIS-QOL 8-item index consists of an eight-item questionnaire with five-point response scales and provides a generic measurement of subjective QoL. Total scores range from 8 to 40, with higher scores indicating better QoL [20]; in this study, we used the means of total scores (range 1 to 5). The EUROHIS-QOL 8-item index was derived from two questionnaires of the World Health Organization Quality of Life (WHOQOL): the WHOQOL-100 [29] and the WHOQOL-BREF [30]. Its domains include overall QoL, general health, energy, daily life activities, self-esteem, personal relationships, finances, and household. The index has been applied in a range of contexts and has been validated in several European countries with good reliability and Cronbach’s alpha value of 0.78 [31].

#### Psychological distress

Psychological distress was measured by the five-item Mental Health Inventory (MHI-5), a subscale of the 36-Item Short Form Health Survey (SF-36) [32]. The MHI-5 is a valid tool for detecting depressive symptoms [33, 34], with Cronbach’s alpha values from 0.74 [33] and includes both positive and negative aspects of mental health. The MHI-5 is recommended for use as an indicator of psychological distress and includes anxiety- and depression-related states [35]. The MHI-5 scores ranged from 0 (poor mental health) to 100 (better mental health). Several cut-off points have been used in primary care settings or certain patient groups. We used a cut-off score of 52, as studies have shown that individuals scoring 52 or less are more likely to suffer from depression [36, 37].

### Predictors

#### Exposure to violence

Respondents were asked, “Has anyone behaved violently towards you over the past 12 months?” Women who reported “obstruction of movement, crabbing, holding, pushing or shoving”, “slapping”, and/or “hitting, kicking, strangling or using a weapon” were categorised as having been exposed to physical violence. “Threat of physical harm either by email or by text message, or in person” was categorised as exposure to emotional violence. “Forced sexual intercourse”, “forced sexual activity”, and “attempt at forced sexual intercourse or other sexual activity” were categorised as exposure to sexual violence. We coded respondents as violence victims if they answered “Yes” to one or more of these three types of violence. If respondents answered “No” to all three, they were coded as unexposed.

Due to considerable multicollinearity and the number of sexual violence victims, types of violence were classified into three mutually exclusive categories. The first of these was physical violence alone or sexual violence alone, the second emotional violence alone, and the third combinations of all three violence types (two or three of physical, sexual or emotional violence).

#### Violence in close relationships

Respondents were asked, “Who used these violence behaviours?” They were given the following response options: “no one”, “unknown person or casual acquaintance”, “present spouse, cohabitee or partner”, or “other person well known to you including ex-spouse”. We coded women who responded “no one” as non-exposed subjects, and women who responded “present spouse, cohabitee or partner” and “other person well known to you including ex-spouse” as victims of violence in close relationships. Our focus is violence in close relationships, which involves not only intimate partners (current or ex) but also other close relationships with e.g. family members or friends.

Therefore, respondents who had been exposed to violence from an “unknown person or casual acquaintance” were coded victims of violence by strangers. We included women who had been exposed to violence both by someone in a close relationship and by a stranger. However, women were excluded from the main analysis if they had been exposed solely to violence from a stranger. In a separate sensitivity analysis we examined the association between violence and QoL for this group.

#### Social characteristics

The available variables relating to social characteristics included age, education, marital status and employment status. Age was classified in three brackets: 19–30, 31–40, and 41–54 years. Educational status was classified in three brackets according to length of education: 9–10 years, 11–14 years, and 15+ years. Marital status was dichotomised as married (in a registered relationship or cohabiting) or non-married, meaning not living with a partner (separated or divorced, widowed, or single). Employment status was dichotomised as employed (full-time or part-time) or not employed (disability pension or recipient of rehabilitation allowance, unemployed or laid off, on family leave or stay-at-home parent, or student).

#### Substance use

There is consistent evidence that substance abuse, heavy drinking and illicit drug use are associated with both perpetration and victimisation of violence in close relationships [38, 39]. Thus, we used alcohol consumption risk and cannabis use as predictor variables.

Alcohol consumption risk was measured with the Alcohol Use Disorders Identification Test - Consumption (AUDIT-C). The AUDIT-C is a widely used screening tool for hazardous alcohol use and consists of three items: frequency of drinking, quantity consumed on a typical occasion, and frequency of heavy episodic drinking [40]. The AUDIT-C is scored on a scale of 0–12, with ≥5 for women indicating at-risk drinking [41]. Although cut-off scores differ according to gender, country or target age group, as score of ≥5 has been used for women aged 20–64 years in Finland [42]. Thus, in this study, AUDIT-C scores were classified in the three groups 0–2, 3–4, and 5+. The number of observations is similar in both groups 0–2 and 3–4. The reference group was set as the middle group 3–4, since it is regarded as a safe level for an adult female population.

Respondents were also asked about their use of cannabis over the past 12 months, and responses were dichotomised as “No” (never used or no) and “Yes”.

### Data analysis

Descriptive statistics (proportions or mean and standard deviation) were used to compare social characteristics, types of violence, QoL, and psychological distress between the violence victims and the group unexposed to violence. The Chi-squared test was used to examine the association between group assignment (unexposed vs. violence victims) and the categorical variables. The independent samples T-test was used to test mean differences in the EUROHIS-QOL 8-item index score between groups (unexposed vs. victims of violence by someone in a close relationship, and victims of violence by someone in a close relationship vs. victims of violence by a stranger).

Ordinary least squares (OLS) regression models were used to test the hypothesis of EUROHIS-QOL 8-item index differences according to the types of violence. Model 1 included predictor variables, and the different types of violence were included as dummy variables. A negative estimate indicates a reduction in QoL. Model 2 introduced social characteristics (age, marital status, education, employment status) as predictor variables. Model 3 introduced substance use (excessive alcohol consumption and cannabis use) as explanatory variables.

The degree of psychological distress among victims of different types of violence was calculated using multivariate logistic regression. Thus, the resulting odds ratios (ORs) with 95% confidence intervals (CIs) indicate over-representation of psychological distress in the victim group. We adjusted the multivariate logistic regressions for social characteristics (model 5) and social characteristics and substance use (model 6). Women who had not been exposed to any type of violence were used as the reference group for all models.

The robustness of the results was checked with two sensitivity analyses. In one, the respondents with the lowest QoL (outliers) were excluded; in the second, each type of violence was analysed separately.

All statistical analyses were conducted using Stata MP Version 15. A *P*-value of 0.05 was considered as the threshold for statistical significance.

## Results

In the ATH survey, 1563 of 20,548 female respondents (7.6%) reported exposure to some form of violence in close relationships during the past year.

Table 1 shows the descriptive statistics for all variables in the models. Women who had been exposed to violence were on average younger (mean age 36.49 vs. 38.72; *p* < 0.001), less educated, less frequently employed, more often single, at higher risk of excessive alcohol consumption (mean AUDIT-C score 3.84 vs. 3.11; p < 0.001), and more likely to have used cannabis during the past year. The mean score of the EUROHIS-QOL 8-item index was significantly lower among violence victims compared to unexposed respondents (3.57 vs. 3.97, *p* < 0.001). A higher prevalence of psychological distress was found among violence victims (30.3% compared to 12.8% among those who had been unexposed).

**Table 1:** Characteristics of women unexposed and exposed to any type of violence in close relationships during the past year

Violence	Overall	Unexposed	Exposed	*p*-value
N	20,548	18,985	1563	
Age in years (%)				< 0.001^c^
19–30	27.0	26.5	33.6	
31–40	26.8	26.6	28.3	
41–54	46.2	46.9	38.1	
Education (%)				< 0.001^c^
Less than 11 years	5.1	5.0	5.7	
11–14 years	33.7	33.2	39.2	
at least 15 years	61.2	61.8	55.1	
Marital status (%)				< 0.001^c^
Married or cohabiting	73.1	74.2	60.5	
Unmarried or no relationship	26.9	25.9	39.5	
Employment status (%)				
Employed	69.1	69.5	64.6	< 0.001^c^
Not employed	30.9	30.5	35.4	
Types of violence (%)				
Physical violence only		–	44.2	
Sexual violence only		–	3.8	
Emotional violence only		–	16.7	
Combinations of violence		–	35.3	
Alcohol risk consumption (AUDIT-C) ^a^ (%)				< 0.001^c^
0–2	39.2	39.8	32.1	
3–4	37.6	38.0	32.6	
5+	23.2	22.2	35.3	
Cannabis use (%)				< 0.001^c^
No	97.3	97.8	92.3	
Yes	2.7	2.3	7.7	
Psychological distress (MHI-5) ^b^ (%)				< 0.001^c^
No	85.9	87.2	69.7	
Yes	14.1	12.8	30.3	
EUROHIS-QOL 8-item index				
mean (sd)	3.94 (0.63)	3.97 (0.62)	3.57 (0.73)	< 0.001^d^

^a^AUDIT-C scores: 5+ indicates excessive alcohol consumption

^b^MHI-5 score of 52 or below indicates psychological distress

^c^Chi-squared test. ^d^Independent-samples t-test

NOTE: The higher the score, the better the QoL (range 1–5)

The results of the OLS regressions, with the mean score of the EUROHIS-QOL 8-item index as the dependent variable and types of violence as explanatory variables (model 1), are shown in Table 2. Non-exposed was the reference. The QoL among victims of all types of violence was statistically significantly lower than among non-victims. Women with combinations of different types of violence had a lower QoL score compared to victims of one type of violence alone. After the introduction of social characteristics (model 2) and social characteristics and substance use (model 3), the estimated differences in QoL decreased, particularly for combinations of violence. However, all estimated parameters remained statistically significant (*p* < 0.01). The model fit, R-squared, improved from 0.03 to 0.09.

**Table 2:** OLS regression of the mean score of the EUROHIS-QOL 8-item index and exposure to different types of violence

	Model 1		Model 2		Model 3	
	est	95%CI	est	95%CI	Est	95%CI
Types of violence Non-exposed	ref.		ref.		ref.	
Physical or Sexual violence only	−0.34***	(− 0.39;-0.30)	− 0.34***	(− 0.38;-0.29)	− 0.32***	(− 0.37;− 0.28)
Emotional violence only	-0.28***	(− 0.36;-0.20)	− 0.24***	(− 0.32;-0.16)	−0.23***	(− 0.31;-0.15)
Combinations of violence	−0.51***	(− 0.57;-0.46)	−0.45***	(− 0.50;-0.39)	−0.44***	(− 0.49;-0.38)
Age in years						
19–30			0.08***	(0.05;0.10)	0.09***	(0.06;0.11)
31–40			ref.		ref.	
41–54			−0.03**	(−0.05;-0.01)	−0.03**	(− 0.05;-0.01)
Education						
Less than 10 years			−0.13***	(−0.17;-0.08)	− 0.12***	(− 0.17;-0.08)
11–14 years			ref.		ref.	
15 years +			0.13***	(0.12;0.15)	0.13***	(0.11;0.15)
Marital status						
Married or cohabiting			ref.		ref.	
Unmarried or no relationship			−0.18***	(−0.20;-0.16)	−0.17***	(− 0.19;-0.15)
Employment status						
Employed			ref.		ref.	
Not employed			−0.22***	(−0.24;-0.20)	− 0.22***	(− 0.24;-0.20)
Alcohol risk consumption (AUDIT-C) ^a^						
0–2					−0.04***	(−0.06;-0.02)
3–4					ref.	
5+					−0.08***	(−0.10;-0.05)
Cannabis use						
No					ref.	
Yes					−0.13***	(−0.19;-0.08)
Adjusted R^2^	0.03		0.09		0.09	

^a^AUDIT-C scores: 5+ indicates excessive alcohol consumption

Model 1: Types of violence only

Model 2: Adjusted for social characteristics

Model 3: Adjusted for social characteristics and substance use

est: parameter estimates. 95%CI: 95% confidence interval

*p < 0.05 **p < 0.01 ***p < 0.001

Experience of a high level of psychological distress was more likely in victims of all types of violence compared to women unexposed to violence (Table 3). The odds ratio of the three different categories of violence were at a similar level, after adjustment for social characteristic and substance-use variables. However, the magnitude of the associations was highest for victims of combinations of violence (OR 4.16; 95% CI 3.44–5.03). After adjusting for social characteristics and substance use, the association remained strong (OR 3.30; 95% CI 2.71–4.02).

**Table 3:** Multivariate logistic regression models of associations of exposure to different types of violence with psychological distress ^a^

	Model 4		Model 5		Model 6	
	OR	(95%CI)	OR	(95%CI)	OR	(95%CI)
Types of violence Non-exposed	ref.		ref.		ref.	
Physical or Sexual violence only	2.46***	(2.06;2.94)	2.38***	(1,99;2,86)	2.25***	(1.87;2.70)
Emotional violence only	2.35***	(1.73;3.18)	2.09***	(1.53;2.85)	2.06***	(1.51;2.81)
Combinations of violence	4.16***	(3.44;5.03)	3.50***	(2.88;4.25)	3.30***	(2.71;4.02)
Age in years						
19–30			1.23***	(1.09;1.38)	1.14*	(1.01;1.29)
31–40			ref.		ref.	
41–54			1.02	(0.91;1.14)	1.02	(0.91;1.14)
Education						
Less than 10 years			1.34**	(1.11;1.62)	1.33**	(1.10;1.61)
11–14 years			ref.		ref.	
15 years +			0.85***	(0.78;0.93)	0.86**	(0.79;0.95)
Marital status						
Married or cohabiting			ref.		ref.	
Unmarried or no relationship			1.79***	(1.63;1.95)	1.71***	(1.56;1.87)
Employment status						
Employed			ref.		ref.	
Not employed			1.53***	(1.40;1.68)	1.57***	(1.43;1.73)
Alcohol risk consumption (AUDIT-C) ^b^						
0–2					0.86**	(0.78;0.96)
3–4					ref.	
5+					1.30***	(1.17;1.45)
Cannabis use						
No					ref.	
Yes					1.44**	(1.16;1.79)

^a^MHI-5 score of 52 or below indicates psychological distress

^b^AUDIT-C scores: 5+ indicates excessive alcohol consumption

Model 4: Types of violence only

Model 5: Adjusted for social characteristics

Model 6: Adjusted for social characteristics and substance use

OR: odds ratio

95%CI: 95% confidence interval

*p < 0.05 **p < 0.01 ***p < 0.001

An independent-samples t-test was performed to compare the mean scores of the EUROHIS-QOL 8-item index between victims of violence in a close relationship and victims of violence by a stranger (data not shown). A total of 85 victims who reported both types of violence were excluded from the analysis. Victims of violence in a close relationship had statistically significant lower mean scores of the EUROHIS-QOL 8-item index than victims of violence by a stranger (3.58 vs. 3.74; *p* < 0.001). All other sensitivity analyses rendered results similar to the primary analysis (results not shown).

## Discussion

The primary aim of this study was to examine the association between exposure to violence over the past 12 months with QoL and psychological distress among women who reported violence in close relationships compared to women who had not experienced violence.

The results of our study showed that 7.6% of women have experienced some type of violence in close relationships in recent years. Exposure to physical violence alone was the most common form of violence. Violence victims were more likely to be younger, have low or no education, and be single [15, 19, 43]. Moreover, cannabis use and excessive alcohol consumption were more common in violence victims. These findings are consistent with earlier findings in other research [44, 45].

We found similar patterns for QoL and psychosocial distress. Our results using both generic QoL measures and mental health instruments fit well with the results from previous studies evaluating effects on QoL and mental health separately [43, 46, 47]. A recent Spanish study also found that current physical or sexual violence was strongly associated with worse health outcomes, current emotional violence being the next strongest association [48].

One of the most important findings was that combinations of violence types had a stronger association with lower QoL and greater psychological distress than exposure to a single type. The scarcity of research in this area may reflect the challenges of conceptualising QoL in relation to the experience of violence, particularly in defining emotional violence [49]. Violence is a complex phenomenon, and different types of violence impact negatively on different dimensions of well-being.

Our focus was not only on violence by intimate partners, but also by perpetrators in other close relationships. The mean score of QoL, as measured by the EUROHIS-QOL 8-item index, was significantly lower among victims of violence in close relationships compared with victims of violence committed by strangers. The definition of “family” or “partner” is quite challenging nowadays and means different things in different cultures. Our focus on ‘close relationships’ allowed us to assess the effects of violence between people who are significant to each other.

Although the number of studies using a narrow definition of violence in close relationships (e.g., current partner) has increased, little is known on the association between QoL and different victim-perpetrator relationships. Our findings suggest that we need a different strategy to prevent or intervene early against violence in close relationships, since it seems to be more pervasive and have more wide-ranging consequences than violence committed by strangers.

The main strength of this study is that we had a large, nationally representative, population-based sample with a relatively high response rate, rather than observations from small populations in clinical settings. Thus, our study provides a relatively complete picture of violence victims compared to other studies, since victims might not disclose experiences of violence to public institutions but may have reported them in a self-administered survey.

All measurement variable scales (EUROHIS-QOL 8-item, MHI-5 and AUDIT-C) used in this study have been validated in previous studies [33, 34, 40]. To the best of our knowledge, several studies have applied the WHO-BREF [50, 51], whereas no study has used the EUROHIS-QOL 8-item index among violence victims. Due to its brevity, we assumed that it might be difficult to capture strong associations between violence and mental health, but our results seem to indicate that the EUROHIS-QOL 8-item index is useful for assessing associations between violence and QoL.

We included substance use (cannabis use and excessive alcohol consumption) as important control variables, because violence exposure is associated with substance abuse [38, 44, 52]. Our results highlighted that violence victims had both high alcohol consumption and cannabis use compared to women unexposed to recent violence.

Available studies tend to focus on the association between physical or sexual violence and QoL [15, 47]. However, our study also included emotional violence and combinations of violence using a variety of violence-related questions.

Despite these strengths, there are several limitations to our study. Since we used cross-sectional data, it is difficult to investigate any causal relationship between violence, mental health and substance abuse. The consequences of violence include negative impacts on mental health, but existing mental health conditions could exacerbate the risk of victimisation.

Secondly, the association between illicit drug use or heavy drinking and violence in close relationships is complex and bidirectional [52]. Substance abuse also contributes to violence exposure, and women exposed to violence may use illicit drugs to self-medicate [53]. However, our results reinforce the evidence of an association between violence victims and substance abuse.

Lastly, we were unable to examine the duration of the violence experienced or the long-term influence of violence on health status. Some women might have experienced violence or poor mental health for more prolonged periods than the last 12 months. Thus, we cannot put forward any causal relationship between lower QoL and violence.

## Conclusions

The results of our study highlight that victims of any type of violence in close relationships have a worse QoL and higher levels of psychological distress than non-victims. These findings provide useful evidence that the complexities of close-relationship violence constitute a serious public health problem. In addition, our results underline that violence is not only a mental health issue but also interacts with victims’ subjective well-being. Some of the differences in QoL are driven by the perpetrator being in a close relationship.

Thus, our findings have important policy implications for developing different preventive strategies according to the victim-perpetrator relationship. Early detection of violence in close relationships needs urgent action in primary health care settings to improve the QoL and prevent severe mental health consequences among women. Our findings could also apply to health education among adolescents, which is pivotal to preventing violence, psychosocial distress, and substance abuse.

## Data Availability

The raw data that support the findings of this study are not publicly available due to participant privacy requirements and the European general data protection regulation. Data from which personal information have been eliminated may be disclosed for research purposes from the Health and Social Data Permit Authority (Findata) in return for a research proposal and an approved user authorisation application.

## Abbreviations

ATH: The Regional Health and Well-being Study
AUDIT-C: Alcohol Use Disorders Identification Test - Consumption
CI: Confidence interval
MHI-5: The five-item Mental Health Inventory
OLS: Ordinary least squares
OR: Odds ratio
QoL: Quality of Life
